# Texture Analysis in Musculoskeletal Ultrasonography: A Systematic Review

**DOI:** 10.3390/diagnostics15050524

**Published:** 2025-02-21

**Authors:** Yih-Kuen Jan, Isabella Yu-Ju Hung, W. Catherine Cheung

**Affiliations:** 1 Department of Health and Kinesiology, University of Illinois at Urbana-Champaign, Urbana, IL 61801, USA; 2 Department of Nursing, Chung Hwa University of Medical Technology, Tainan 717, Taiwan; isabella@ms.hwai.edu.tw; 3 Doctor of Physical Therapy Program, Northern Illinois University, DeKalb, IL 60115, USA; wcheung@niu.edu

**Keywords:** gray-level co-occurrence matrix, gray-level run-length matrix, skeletal muscle, texture feature, ultrasound

## Abstract

**Background:** The objective of this systematic review was to summarize the findings of texture analyses of musculoskeletal ultrasound images and synthesize the information to facilitate the use of texture analysis on assessing skeletal muscle quality in various pathophysiological conditions. **Methods:** Medline, PubMed, Scopus, Web of Science, and Cochrane databases were searched from their inception until January 2025 using the PRISMA Diagnostic Test Accuracy and was registered at PROSPERO CRD42025636613. Information related to patients, interventions, ultrasound settings, texture analyses, muscles, and findings were extracted. The quality of evidence was evaluated using QUADAS-2. **Results:** A total of 38 studies using second-order and higher-order texture analysis met the criteria. The results indicated that no studies used an established reference standard (histopathology) to evaluate the accuracy of ultrasound texture analysis in diagnosing muscle quality. Alternative reference standards were compared, including various physiological, pathological, and pre–post intervention comparisons using over 200+ texture features of various muscles on diverse pathophysiological conditions. **Conclusions:** The findings of these included studies demonstrating that ultrasound texture analysis was able to discriminate changes in muscle quality using texture analysis between patients with pathological conditions and healthy conditions, including popular gray-level co-occurrence matrix (GLCM)-based contrast, correlation, energy, entropy, and homogeneity. Studies also demonstrated that texture analysis can discriminate muscle quality in various muscles under pathophysiological conditions although evidence is low because of bias in subject recruitment and lack of comparison with the established reference standard. This is the first systematic review of the use of texture analysis of musculoskeletal ultrasonography in assessing muscle quality in various muscles under diverse pathophysiological conditions.

## 1. Introduction

Musculoskeletal impairment is the leading cause of disability and rehabilitation in the world [1]. Medical imaging-based diagnosis and monitoring are essential for effective musculoskeletal rehabilitation [2]. B-mode ultrasound is a low-cost, non-invasive, portable medical imaging modality that can be used to improve musculoskeletal rehabilitation [2]. Ultrasound assessment of the musculoskeletal system usually focuses on structural and morphological information, such as the thickness, cross-sectional area, and regional abnormality (e.g., tears and trigger points). In addition, echo intensity of ultrasound images has been increasingly used as an indirect index to assess muscle composition, for example, sarcopenia [2,3]. However, variations in ultrasound measurements are observed due to the operator’s experience and ultrasound settings that limit the generalization of ultrasound examinations in routine rehabilitation practice [3]. Although machine learning and deep learning techniques have shown promise in improving ultrasound diagnosis, these techniques may need time to be adopted by ultrasound industry [4].

Texture analysis of ultrasound images has been demonstrated to effectively extract complex spatial features hidden from visual observations of musculoskeletal ultrasound images [3,5]. Texture analysis involves the mathematical and statistical representations of pixel intensity and spatial distribution within the designated region of interest (ROI) [6,7]. These features can be utilized to describe spatial patterns related to the muscle composition and quality, reflecting the influences of various pathophysiological conditions or therapeutic interventions [3]. Ultrasound texture analysis has been demonstrated to minimize the influence of the operator’s experience and ultrasound settings on extracted features of ultrasound images. Texture analysis has three categories including first-order (e.g., echogenicity-related features), second-order (e.g., Haralick’s and Galloway’s features), and higher-order (e.g., Local Binary Pattern) analysis [6,7]. Research demonstrated that second-order texture analysis is less sensitive than the popular echo intensity method [3]. Although texture analysis has been widely applied in various tissues (thyroid cancers and liver fibrosis), ultrasound texture analysis of skeletal muscle has gained much less attention. There are no evaluations and guidelines of texture analyses of muscle ultrasound images. In order to address this gap, the objective of this systematic review is to summarize the findings of texture analyses of musculoskeletal ultrasound images and evaluate evidence and synthesize the results to improve test accuracy for improving patient outcomes. To the best of our knowledge, this is the first systematic review of texture analyses of musculoskeletal ultrasound images.

## 2. Methods

This diagnostic test accuracy review addressed the question of how good ultrasound texture analysis is at assessing changes in skeletal muscle quality from B-mode ultrasound images. The second-order and higher-order texture analyses were included and the first-order texture analysis (echogenicity) was excluded as it does not address the spatial relationship between two or more pixels. This systematic review was conducted in accordance with the Preferred Reporting Items for Systematic Reviews and Meta-analyses for Diagnostic Test Accuracy (PRISMA-DTA) [8] and the Cochrane Handbook for Systematic Reviews of Diagnostic Test Accuracy [9]. The protocol was registered at the International Prospective Register of Systematic Reviews (PROSPERO CRD42025636613).

### 2.1. Search Strategy

The focus of this review is the use of texture analysis (second-order and higher-order methods) of ultrasound as a diagnostic test for assessing skeletal muscle quality. A comprehensive search was performed in the Medline, PubMed, Scopus, Web of Science, and Cochrane databases up to January 2025. The search strategy included the following terms: (ultrasound OR ultrasonography) AND (muscle OR skeletal muscle NOT cardiac) AND humans AND texture.

### 2.2. Eligibility Criteria

The PICOS framework (Population, Intervention, Comparison, Outcome, Study design) was used to determine the eligibility of the study. (1) The population included all individuals of all ages and ethnicities. Both patients and healthy individuals were included. The target in this review was the skeletal muscle. (2) Intervention included all interventions and no interventions. (3) Comparison included the reference standard, pre–post intervention comparisons, and between-groups comparisons. (4) Outcome included texture features of musculoskeletal ultrasound. Texture features refer to the second-order and higher-order methods. First-order texture features, such as echogenicity, were excluded in this systematic review because the first-order texture feature does not address the spatial patterns between pixels of the muscle ultrasound image. (5) The study design included all study designs in human beings with at least 3 participants in each group. Both prospective and retrospective studies were included. Secondary analyses were included if a new texture feature was investigated. A study using multiple ultrasound devices was excluded. Studies using phantom investigation or computational simulation without real human data were excluded. Studies that used texture analysis to segment two tissues (e.g., breast and muscle tissues) without discussing muscle texture were excluded.

The index test of the PIT framework (Population, Index test, Target condition) was also used to determine the eligibility of potential studies. The index test was a texture analysis of B-mode ultrasound images. Other forms of ultrasound imaging were excluded, such as radiofrequency ultrasound, shear wave ultrasound, strain elastography, and high-frequency ultrasound.

### 2.3. Study Selection and Data Extraction

Two reviewers (Y.J. and W.C.) independently screened the titles and abstracts of all relevant studies. Full-text articles were obtained for studies that met the inclusion and exclusion criteria. This review included journal articles published in English and excluded conference papers, reviews, reports, and theses. Duplicates were removed through Endnote (X8, Clarivate, Philadelphia, PA, USA). The journal articles were then evaluated to determine the final inclusion. Any disagreements were solved through discussions or a third reviewer (I.H.). The data collection process was performed by two reviewers using the developed form. The items on the form included the study (authors and year), patients, interventions, ultrasound image acquisition, reference standard, type of muscles, region of interest, texture features, and findings.

### 2.4. Quality Assessment

Quality assessment was undertaken using the Quality Assessment Tool for Diagnostic Accuracy Studies 2 (QUADAS-2) [10] which included 7 points: (1) bias in patient selection, (2) concerns regarding patient applicability, (3) bias in index test interpretation, (4) concerns regarding index text applicability, (5) bias in reference standard, (6) concerns regarding reference standard applicability, and (7) concerns regarding flow and timing. The reference standard is defined as the gold standard for clinical practice. If this is absent from the research, an alternative reference can be obtained through the case–control study design by comparing texture features between patients and controls. Assessments were performed by two reviewers (Y.J. and W.C.) and any disagreement was resolved with the third reviewer (I.H.). The reporting bias was not investigated because of the lack of appropriate statistical methods for the diagnostic accuracy test.

### 2.5. Diagnostic Accuracy Measures and Synthesis Methods

In this review, accuracy is defined as the ability of ultrasound texture analysis to discriminate changes in muscle quality from B-mode ultrasound images in various pathophysiological conditions, for example, age, gender, type of muscles, post-exercise, post-rehabilitation, and neuromuscular diseases. If a texture feature is able to discriminate the change in muscle quality from ultrasound images, it is a positive test result. The reference standards are muscle biopsies, magnetic resonance imaging (MRI), or biomarkers justified by the author. The synthesis method was based on predetermined topics of medical imaging research including: (1) current state of ultrasound texture analysis, (2) image acquisition, (3) image segmentation, (4) feature extraction, (5) image interpretation, (6) clinical implications including the benefits and harms of ultrasound texture analysis. Under these themes, a narrative synthesis was conducted to evaluate evidence and guide clinical practice and research.

## 3. Results

### 3.1. Study Selection

**Figure 1:**
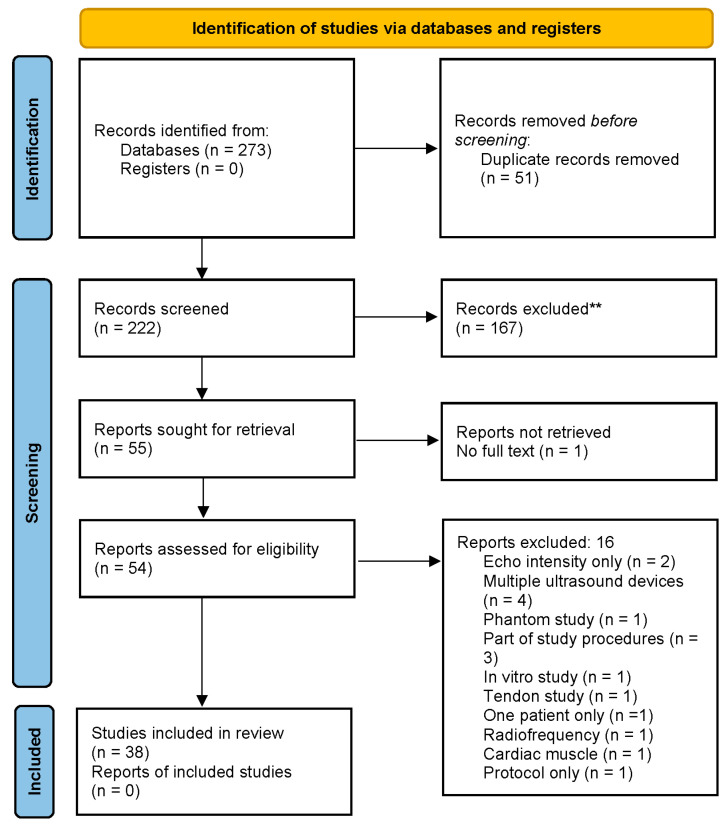
PRISMA Flow diagram.

The initial search identified 273 relevant articles. After removing the duplicates, the total number was reduced to 222 studies. Based on the title and abstract, 54 full-length articles were retrieved for the eligibility assessment, of which 38 articles met the inclusion and exclusion criteria and were included in this systematic review (Figure 1). The PRISMA checklist and the list of excluded studies and reasons are provided in the Supplemental Files.

### 3.2. Study Characteristics

The full details of the characteristics of the included studies are provided in the Table 1. (population, ultrasound information, region of interest related information, and findings).

For the publication year, the earliest study included in this review was published in 2006 [11] followed by 2013 [12], 2015 [13,14], 2016 [15], 2017 [16,17,18,19], 2018 [20,21,22,23], 2019 [24,25,26,27,28], 2020 [29,30,31,32], 2021 [33,34], 2022 [35,36,37,38], 2023 [39,40,41,42], and 2024 [43,44,45,46,47,48].

For the type of muscles, there was the abductor pollicis brevis [28], biceps brachii [13,14,17,20,26,31,36,45,46,47], brachialis [16,22,36], brachioradialis [46], extensor carpi radialis [46], forearm flexors [17], gastrocnemius [14,16,18,23,26,27], hamstring [41], quadriceps [11,14,17,19,26,32,34,35,36,37,38,40,43,47,48], rectus abdominis [35], supraspinatus [11,15,25], tibialis anterior [14,17,26], trapezius [12,21,24,29,30,33,39,42], and triceps brachii [44].

A variety of texture features have been found from the included studies, including:

(1) Haralick’s features (based on the gray-level co-occurrence matrix, GLCM): 10+ features [13,18,23,24,25,27,29,37,39,40] or (cluster prominence [15], cluster shade [15], contrast [14,15,17,21,22,28,32,34,35,36,38,41,42,44,45,47,48], correlation [14,15,17,21,22,28,32,34,35,36,38,41,42,43,44,47,48], energy/uniformity/angular second moment [14,15,17,19,28,32,34,35,36,41,42,44,45,47,48], GLCM-based entropy [14,15,17,19,21,28,34,38,41,42,45,47,48], homogeneity/inverse difference moment [14,15,17,19,21,28,34,35,36,38,41,42,43,44,45,47,48], inertia [15], and symmetry [14]).
(2) Galloway’s features (based on the gray-level run-length matrix, GLRM): 8+ features [18,23,24,27,29,37,39,40] or (short-run emphasis (SRE) [14,21], long run emphasis (LRE) [14,21], run-length distribution normalized by ROI (RLN) [21], run percentage (RP) [14,21], gray-level distribution normalized by ROI (GLN) [21], low gray-level run emphasis (LGRE) [21], high gray-level run emphasis (HGRE) [21], gray-level non-uniformity (GLNU) [14], and run length non-uniformity (RLNU) [14]).
(3) Local binary pattern [14,21,30,32,35,38,39,42].
(4) Blob analysis [11,21,30,33,35,39].
(5) Texture anisotropy index [20,46].
(6) Entropy-based methods: entropy filter [12] and 2D entropy [31].
(7) Complexity curve-based methods (maximum value of transitions, averaged value of transitions, sample mean of the gray-level value weighted by the number of transitions, sample standard deviation of the gray-level value weighted by the number of transitions, and entropy) [16].
(8) Model-based methods: autoregressive model [18,23].
(9) Transform-based methods: wavelet-based features [13,18,23], scale invariant feature transform [26], and Gabor transform [42].

Comparisons of texture features included the following: (1) ultrasound settings (ultrasound frequency and ROI size), (2) physiological comparisons (age, sex, muscle types, and areas within a muscle), (3) pathological conditions, (4) pre- and post-interventions, (5) accuracy of classification, e.g., machine learning models and statistical approaches, and (6) reference standards.

(1) Ultrasound settings: resolution/ultrasound frequency [32,35], gain [20], and ROI sizes [20].
(2) Physiological conditions: age [19,23,37,38,40,47], areas within a muscle [41], muscle types [11,16], temporal variations [16], and sex/gender [14,19,26,43].
(3) Pathological conditions: amyotrophic lateral sclerosis [17,28], breast cancer [36], chronic kidney disease [34,48], diabetes mellitus and prediabetes [35], Duchenne muscular dystrophy [20,46], fibromyalgia [29], muscle inflammation [15], muscle tear [15,25], myofascial pain [12,21,24,30,33,39,42], myogenic diseases [18], myositis [13,27], myotonic dystrophy [27], neurogenic diseases [18], and spinal muscular atrophy [46].
(4) Pre- and post-interventions: aerobic exercise [36], cupping therapy [44], isometric exercise (20, 30, 40, 50% of maximal voluntary contraction) [31], and eccentric exercise-induced muscle damage [22,45].
(5) Reference standards and accuracy/classification related outcomes:
  a. 3T MRI on the muscle-fat percent of the hamstring and quadriceps (GLCM energy (β = 0.57) and GLCM homogeneity (β = 0.61)) [48].
  b. Vibration elastography of the trapezius (entropy filter, sensitivity 0.74, specificity 0.81) [12].
  c. Electromyography on muscle fatigue of the biceps (entropy) [31].
  d. Clinicians (orthopedic surgeon: overall accuracy 87.9% and 88.4% for inflammation, 83.3% for calcific tendinitis, and 92.3% for tears of the rotator cuff) [15].
  e. Statistical approaches for the biceps, forearm flexor, quadriceps, tibialis anterior, supraspinatus, gastrocnemius, abductor pollicis bravis [17,25,27,28]: (Sensitivity 76.4 ± 21.9%, specificity to 97.7 ± 1.9%; GLCM area under the curve (AUC) 0.849/0.874/0.977/0.934, sensitivity 0.81/0.79/0.94/0.85, and specificity 0.73/0.83/0.94/0.88 for biceps brachii/forearm flexors/quadriceps/tibialis anterior; AUCs 0.70–0.90 between controls and neurogenic patients, 0.57–0.80 between controls and myogenic patients, and 0.95–1.0 between neurogenic and myogenic patients) [18].
  f. Machine learning and deep learning for the biceps, trapezius, supraspinatus, and vastus lateralis (support vector machine (AUC 0.67–0.79, 85/87% accuracy, 90% sensitivity, and 83/85% specificity; 86.96% accuracy, a Matthews correlation coefficient of 0.724, 88% sensitivity, and 86% specificity) [13,24,29], k-NN [13], Fisher [13], and logic regression [29]).

### 3.3. Quality of the Study

The assessments of the included studies using QUADAS-2 [10] are provided in the Supplemental File. All studies have high bias and concern regarding the patient selection, patient applicability, index test interpretation, and index test applicability. There are no studies following Diagnostic Test Accuracy guidelines with which to assess the accuracy by using one group’s study design with random orders of the reference standard and index test on diagnosing muscle quality. Most studies used the “case–control study” in which the case is the individuals with the known condition in muscle quality and the control is healthy individuals without the known condition. Variations in reference standards (i.e., MRI, elastography, electromyography, physician diagnosis, and physiological conditions) cause inappropriate statistical combining of the numerical data.

## 4. Discussions

Findings from the included studies were grouped into themes including the current state of ultrasound texture analysis, image acquisition, image segmentation, feature extraction, image interpretation, and clinical implications for discussion and synthesis.

### 4.1. Current State of Ultrasound Texture Analysis

A systematic review of diagnostic test accuracy assesses the results of an index test against a reference standard. In this review, no studies have conducted a typical diagnostic test to evaluate the accuracy of ultrasound texture analysis against a validated diagnosis, and a variety of alternative designs were used to explore the use of texture analysis for assessing muscle quality. This reflects insufficient evidence on ultrasound texture analysis as a clinical tool on assessing muscle quality. However, the results mapped by this systematic review indicate that texture analysis shows promise in discriminating changes in muscle quality in various conditions ranging from physiological conditions (age [19,23,37,38,40,47], spatial variations within a muscle [41], muscle types [11,16], temporal variations [16], and sex [14,19,26,43]), pathological conditions (amyotrophic lateral sclerosis [17,28], Duchenne muscular dystrophy [20,46], and myofascial pain [12,21,24,30,33,39,42]), and pre–post intervention comparisons (aerobic exercise [36], cupping therapy [44], isometric exercise [31], and eccentric exercise [22,45]). The findings of these studies provide a foundation to support the use of ultrasound texture analysis for assessing muscle quality in various pathophysiological conditions. Nevertheless, there is a need to validate the ultrasound texture analysis against an established assessment for assessing muscle quality. A standard design for a test accuracy study is a single group of consecutive participants using a cross-sectional study design that indicates how well the test performs in identifying patients at the time of testing, not whether the patients will develop (or developed) the target condition in the future (or past) [9,10]. In this case, it does not matter which test (ultrasound texture analysis or the reference standard) is conducted first.

Regarding the clinical pathway, ultrasound texture analysis does not have a competing index for its proposed role as an accessible diagnostic test in muscle quality in rehabilitation settings. Other existing tests, such as muscle biopsies and MRI, may not be suitable for the long-term daily routine monitoring of rehabilitation progression because of their invasive or radiation effects and much higher costs compared to diagnostic B-mode ultrasound. Although shear wave elastography may be a promising tool for assessing muscle quality, it is unclear whether the shear modulus measured from shear wave elastography could represent muscle quality. Thus, texture analysis of B-mode ultrasound images is recommended for all target populations who need muscle quality assessment. The values of texture features can provide feedback to patients and clinicians to monitor the progression of rehabilitation interventions.

### 4.2. Image Acquisition

Various B-mode ultrasound devices have been used in the included studies and are listed in Table 1. A linear-array transducer with multi-frequency ranges (~6–15 MHz) was usually used to acquire muscle ultrasound images. Most studies kept the ultrasound settings (gain, ultrasound frequency and time-gain compensation) the same across all participants. Some studies selected different ultrasound frequencies to accommodate the thickness of larger muscles. The measured depth of ultrasound should cover the muscle from the skin surface to the bony area. Research studies have demonstrated that the ultrasound settings (e.g., ultrasound frequency) should be kept the same across the participants [32]. Histogram-based analyses, such as echovariation, skewness, and kurtosis, are particularly influenced by the ultrasound settings [3]. Most studies did not report the image format (e.g., Digital Imaging and Communications in Medicine (DICOM)). Current evidence appears to support the use of all listed commercial ultrasound devices for muscle quality assessment.

### 4.3. Image Segmentation

Most studies used visual observations and manual segmentation to determine ROI within the muscle. Some studies selected a relatively small ROI due to the limitation of the ultrasound device and most studies chose an ROI that was as large as possible to fully capture the texture features of the muscle [4]. Theoretically, a large ROI should be selected to cover the whole muscle to fully capture muscle quality and a small ROI may be influenced by the spatial heterogeneity of biological tissues, including the skeletal muscle [13,44]. Dubois et al. compared different sizes of ROI within a muscle and found that ROI sizes affected texture feature values [20]. Sahinis and Kellis compared different locations of the hamstring muscle and found significant differences in texture feature values at different locations of the hamstring [41]. The shape of ROI used in included studies ranged from a single rectangular and circular area to multiple circular areas without clear justifications. In addition, when identifying the edge of the muscle, the subcutaneous fat tissue over the muscle can significantly attenuate the ultrasound energy. This is particularly challenging when the fat filtration is significantly increased due to aging and pathological conditions. This indicates a need to standardize the location for assessing a muscle as well as a need to cover a large area or multiple areas of a muscle.

### 4.4. Feature Extraction

B-mode ultrasound is a 2D image, which may be challenging for clinicians to use to describe the change in spatial patterns of the muscle following a pathophysiological condition. However, spatial patterns of the whole muscle or a small region of interest of muscle ultrasound images can be quantified by texture analysis. Nowadays, free and affordable software packages are available to perform texture analysis, including Matlab and Image Processing Toolbox (The MathWorks, Natick, MA, USA), ImageJ (National Institutes of Health, Bethesda, MD, USA), MaZda (qmazda.p.lodz.pl/), Imaging Biomarker Explorer (IBEX, bit.ly/IBEXSrc_MDAnderson), and LIFEx (lifexsoft.org). It is worth mentioning that different software packages may result in differences in the computed texture feature values due to variations in image preprocessing and algorithm implementation [49]. More research is needed to clarify the differences using various texture analysis packages. The use of a texture analysis software package can yield more than 200+ texture features. There is a need to find the redundancy of texture features and identify the representative texture analysis for various pathophysiological conditions.

The statistical texture methods include the first-order, second-order, and higher-order methods. First-order texture features refer to the histogram-based features, including the mean, standard deviations, kurtosis, and skewness of all pixels of an image. Second-order texture features refer to the spatial relationship of groups of 2 pixels of an image, including GLCM-based Haralick’s features and GLRM-based Galloway’s features. Third-order texture features refer to the spatial relationship of groups of 3 pixels of an image. Ultrasound echo intensity is often used to assess muscle composition changes, which is the first-order texture feature, without addressing the spatial relationship between pixels. The second-order is usually implemented based on the gray-level co-occurrence matrix (GLCM, also known as the gray-level spatial dependence matrix) [7] or gray-level run-length matrix (GLRM) [6]. Higher-order texture features (including nonlinear features) include texture anisotropy index [20,46], blob analysis [11,21,30,33,35,39], and entropy filter [12].

The most popular texture features of muscle ultrasound images are proposed by Haralick [7] to quantify the texture of an ultrasound image by using the ultrasound image to create a GLCM and then extracting statistical texture features. These features are calculated based on a GLCM derived from the original ultrasound image rather than the original image itself. The GLCM of an ultrasound image is an estimate of the statistical probability *P_δ_* (*i*, *j*) of the echo intensity values of two pixels ***i*** and ***j*** at a distance (***δ***) along an angle (***θ***). An eight-bit ultrasound image would result in GLCM with dimensions of 256 × 256. It takes enormous computing to find all GLCMs. Haralick et al. suggested calculating GLCM at ***θ*** = 0°, 45°, 90°, and 135° with ***δ*** at 1 or 2 pixels [7]. Although there are more than 20 features, 5 texture features were commonly used in most studies, including the contrast [14,15,17,21,22,28,32,34,35,36,38,41,42,44,45,47,48], correlation [14,15,17,21,22,28,32,34,35,36,38,41,42,43,44,47,48], energy/uniformity/angular second moment [14,15,17,19,28,32,34,35,36,41,42,44,45,47,48], GLCM-based entropy [14,15,17,19,21,28,34,38,41,42,45,47,48], and homogeneity/inverse difference moment [14,15,17,19,21,28,34,35,36,38,41,42,43,44,45,47,48].

Contrast texture measures the local variations in GLCM derived from the muscle ultrasound image. According to Wilkinson’ study [34], a lower value of contrast texture indicates regions that have a high homogeneity. Correlation texture refers to the joint probability occurrence of the pixel pairs. A greater value of correlation texture indicates that regions have similar gray levels, as sign of high homogeneity. Energy texture provides the sum of squared elements in GLCM. When an ultrasound image has high energy texture, the image has high contrast and sharp transitions. Entropy texture measures the randomness of intensity distribution and entropy is high when pixels’ intensity distributions are irregular. Homogeneity texture measures the closeness of the distribution of elements in GLCM. When an ultrasound image is homogeneous, the value of homogeneity index is higher. Although these Haralick’s features are highly correlated, it is usually recommended to select several texture features to characterize muscle quality because the exact physiological meanings of these texture features have not been well established [3,41,44].

Galloway’s features are based on the gray-level run-length matrix (GLRM) [6] to quantify the texture of an ultrasound image by using the ultrasound image to create GLRM and then extracting statistical textures. In GLRM, the pixel ***R*(*i***, ***j*)** contains the number of pixels with the run length (***j***) and the intensity (***i***) in a given direction. A run length is a set of constant-intensity pixels located in a line. Run length is calculated by counting the number of runs of a given length for each gray level. Galloway’s features are less common compared to Haralick’s features in the included studies, and the underlying physiological meanings require further research.

In addition to the statistical methods (e.g., Haralick’s and Galloway’s features), transform-based, structure-based, and model-based methods are found in the included studies. Transform-based methods denote techniques converting an ultrasound image into a new form using spatial frequency transform methods, such as wavelets [13,18,23] and Gabor [42] filters, for assessing the phase and direction. Structure-based methods describe a texture as the composition of texture elements such as fractal dimension. The use of structure-based methods is limited to describe regular textures. These methods are less common compared to the statistical methods in musculoskeletal ultrasound.

### 4.5. Limitations and Future Research

This systematic review has limitations. A meta-analysis was not performed due to the heterogeneity and differences in reference standards of the included studies. Because of the lack of a gold standard, most studies did not specifically assess the accuracy, sensitivity, and specificity of ultrasound texture features on discriminating muscle quality in various conditions. Some studies evaluated the accuracy of texture analysis against machine learning models. However, these machine learning models are trained by the authors on certain images and their generalization may be poor. Therefore, no definite diagnostic test accuracy could be established from this review. There is a need for further research to assess the accuracy of ultrasound texture analysis on muscle quality using the standard study design for diagnostic test accuracy. Also, this review does not establish the specific physiological meaning for texture features. The included studies were conducted in a variety of conditions including various patients under various interventions to a number of muscles using various texture features. Therefore, the meanings of these texture features cannot be established.

No studies compared the diagnostic accuracy between the reference standards (e.g., muscle biopsy) and texture analyses of muscle ultrasound images in the included studies of this systematic review. Although muscle biopsy has limitations, muscle biopsy remains the gold standard for diagnosing neuromuscular diseases [50,51]. Muscle biopsy techniques for assessing neuromuscular diseases range from needle muscle biopsy to open surgical biopsy. According to a recent systematic review [51], all types of muscle biopsy procedures were well tolerated with few adverse events, and no scarring complications were reported. However, due to its invasive nature, muscle biopsy may not be feasible for routine assessments in rehabilitation and exercise training. For example, the use of daily muscle biopsy may not be appropriate for assessing the efficacy of a rehabilitation intervention on improving muscle quality. However, a texture analysis of muscle ultrasound images can overcome the issue associated with invasive nature of muscle biopsy. The quality assessment performed by this systematic review indicates that all included studies did not use the standard study design suggested by PRISMA Diagnostic Test Accuracy and QUADAS 2.0; therefore, the evidence level and accuracy of ultrasound texture analyses could not be established. Researchers interested in muscle quality assessment should reference the development process of using ultrasound to diagnose thyroid cancer and liver fibrosis [52,53]. Although ultrasound texture analysis has shown promise in discriminating muscle quality in the “case–control study” design (discriminating muscle quality in two groups consisting of patients and healthy controls), more studies are needed to establish its sensitivity and specificity for diverse neuromuscular diseases.

Although no gold standard (histopathology) was compared to texture analysis of muscle ultrasound images, various alternative references were used to demonstrate the ability of texture analysis to quantify pathological changes in muscle quality. In these scenarios, pathophysiological conditions were already diagnosed and the authors compared muscle quality between patients and healthy controls. The results of these studies provide initial guidelines on using ultrasound texture analysis, including spatial variations in muscle quality within a muscle, the muscle type, the gender effect, and ultrasound settings on texture values as well as the physiological meanings of each texture feature in muscle quality assessment. Wilkinson et al. compared the muscle quality of the quadriceps and hamstring between patients with chronic kidney disease and healthy controls using both MRI and ultrasound texture analysis and provided primary evidence that ultrasound texture analysis could be a promising indicator of a reduced percentage of skeletal muscle compared to MRI, a reliable imaging tool. Another study compared the performance of ultrasound texture analysis of the rotator cuff with the diagnosed muscle conditions and demonstrated 88.4% accuracy for inflammation, 83.3% accuracy for calcific tendinitis, and 92.3% accuracy for muscle tears [15]. Other studies used statistical [17,25,27,28] and machine learning [13,24,29] approaches to classify muscle ultrasound images of patients from healthy controls. Although these are all examples of studies on the classification of muscle ultrasound images of various muscles from diverse pathological conditions, these results (sensitivity and specificity) cannot be used as true sensitivity and specificity measures because of the study design bias. To the best of our knowledge, this is the first systematic review based on PRISMA Diagnostic Test Accuracy and QUADAS 2.0 to assess the evidence of ultrasound texture analysis of musculoskeletal images and the findings of this systematic review indicate a need to follow these clinical guidelines to establish the accuracy of ultrasound texture analysis of musculoskeletal images.

Previous studies have demonstrated that pathological conditions are associated with a decrease in homogeneity of the soft tissue including the skin, subcutaneous tissue, and muscle and exercise training can lead to an improvement in homogeneity of the soft tissue [3,22,41]. These positive outcomes also suggest that ultrasound texture analysis can be used to assess bulk soft tissue (consisting of muscle, fat, fascia and skin) at risk of tissue damage. Various neurological (spinal cord injury) and metabolic (diabetes) diseases cause alterations in soft tissue, with an increased risk of tissue damage [5,54]. Assessing deteriorated soft tissue using ultrasound texture analysis could help monitor the risk of tissue damage. Texture analysis has demonstrated its promise in discriminating changes in muscle quality in various muscles under various pathophysiological conditions. Future research should examine the validity of a cluster of texture features in assessing muscle quality in various conditions.

Future research should determine the diagnostic accuracy of the texture analysis musculoskeletal ultrasound in various pathophysiological conditions, including the disease type, age, gender, muscle types, and location within a muscle (proximal, middle, and distal). A narrative review by Paris et al. [3] highlights the potential of using texture analysis of musculoskeletal ultrasound for assessing muscle quality in various pathophysiological conditions. A number of studies have been conducted to explore its potential in assessing muscle quality. In this systematic review, we further evaluate these findings and highlight the findings and future research needed in this topic.

## 5. Conclusions

Musculoskeletal ultrasonography is a promising diagnostic tool for assessing adaptation and pathophysiological remodeling of the skeletal muscle to exercise, interventions, and injury. Common ultrasound assessments focus on the changes in the thickness, length, and cross-sectional area of the skeletal muscle. Recently, echogenicity, a first-order texture feature, has been used to assess muscle composition, while second-order and higher-order texture features have not been widely used to assess muscle quality and composition. This systematic review identified 38 studies exploring the use of second-order and higher-order texture features in discriminating muscle quality in various pathophysiological conditions and found that texture analysis could discriminate muscle quality in various comparisons. These findings support the use of B-mode ultrasound with texture analysis as a routine diagnostic tool in rehabilitation settings to assess the progression of muscle quality after interventions. This review also found that there is insufficient evidence for ultrasound texture analysis and more research is needed to establish its accuracy, sensitivity, and specificity using the standard diagnostic accuracy test guidelines. This systematic review confirms that texture analysis of B-mode muscle ultrasound images is a promising diagnostic tool for assessing muscle quality but that current evidence is insufficient to support ultrasound texture analysis as a validated diagnostic test yet.

Specifically, looking at second- and higher-order texture analysis of musculoskeletal ultrasound, this systematic review found that: (1) texture analysis is a sensitive test to discriminate acute changes following rehabilitation interventions (e.g., eccentric muscle contraction, different percent of maximal muscle contraction, and cupping therapy), differentiate spatial changes in muscle quality of a muscle, effects of the gender, age, ultrasound settings, and software for texture analysis on muscle quality, indicating its potential as a non-invasive diagnostic tool in routine clinical practice; (2) a variety of texture features (200+) are associated with specific pathophysiological conditions, indicating a need to establish the exact physiological meaning of each of texture feature for muscle ultrasound images; and (3) QUADAS-2 reveals a high risk bias on subject recruitment and low applicability of the results, indicating a need to determine the diagnostic accuracy of texture analysis against an established reference standard, such as muscle biopsy.

## Figures and Tables

**Table 1:** Characteristics of included studies.

Study	Patients (Pathophysiological Conditions), Muscles	Image Acquisition, Region of Interest (ROI)	Texture Analyses	Findings
Nielsen, et al. [11]	9 healthy controls (7 females and 2 males) for muscle comparisons (supraspinatus and vastus lateralis, longitudinal, and cross-sectional)	Diasonics VST Masters Series System, GE Medical, 5 (3.5–7) MHz curved linear array probe, depth 5 cm, same settings for all. ROI: 512 × 512 pixels by visual inspection	Echogenicity (first order) and blob analysis (higher order)	The vastus lateralis has more non-contractile components and is coarser than the supraspinatus (*p* < 0.05). Echogenicity analysis complements blob analysis more in assessing muscle composition than the mean gray-scale value alone.
Turo, et al. [12]	14 patients (4 males and 10 females) with myofascial pain (trigger points) and 15 healthy controls (9 males and 6 females) (upper trapezius)	SonixRP US system (Ultrasonix Medical, Vancouver, Canada), 5–14 MHz, depth 2.5 cm, same settings. R01: muscle belly 60% and fascial border 40%, the assessor blinded, color Doppler variance imaging of elastography as the reference	Entropy filter (higher order)	Echotexture analysis using local entropy can distinguish between subjects with painful trigger points and those without pain and myofascial trigger points have significantly lower entropy (*p* < 0.05). Mean entropy vs. color Doppler area has sensitivity 0.74 and specificity 0.81. The combination of both entropy and elastography yielded 60% sensitivity and 81% specificity.
König, et al. [13]	11 patients with myositis and 7 healthy controls; 2 radiologists (biceps brachii, transvers plan)	Toshiba Aplio XG system, 9 MHz linear array transducer: width 50 mm, depth 30 mm, 75% gain, rectangular and polygonal ROI, 60 images	5 first-order (mean, sd, skewness, kurtosis, and entropy of the histogram), 130 wavelet-based and 240 Haralick’s features (d = 1, 2, 3, 4, 5; theta 0, 45, 90, 135) with SVM and k-NN, Fisher	SVM was the best for discriminating healthy and pathological muscle tissue, achieving 85/87% accuracy, 90% sensitivity, and 83/85% specificity, depending on the radiologist. Rectangular ROI is better than polygonal ROI.
Molinari, et al. [14]	20 healthy individuals (10 males and 10 females) for gender effects on muscles (biceps brachii, vastus lateralis, rectus femoris, medial gastrocnemius, tibialis anterior)	MyLab Twice ultrasound (Esaote, Genoa, Italy), 3–13 MHz, 50% gain, depth 44 mm adjusted for each individual, DICOM, 1ROI except quadriceps 2 ROI	53 features: 7 first order (integrated optical density, mean, SD, variance, skewness, kurtosis and energy1), 24 Haralick (symmetry, contrast, homogeneity, entropy, energy2, correlation), 20 Galloway (SRE, LRE, GLNU, RLNU, RP), 2 LBP	Multi-texture analysis may be useful for diagnosing muscle damage and myopathic disorders. Sensitivity of 76.4 ± 21.9%, specificity of 97.7 ± 1.9%. The combination of first-order and higher-order texture features (Haralick, Galloway and LBP) can be used to discriminate gender and muscle types.
Chang, et al. [15]	*Secondary analysis*, 93 subjects (43 males); 99 shoulder ultrasound images including 43 muscle inflammation, 30 calcific tendinitis and 26 muscle tear. (supraspinatus)	Aloka alpha-6 (Hitachi, Tokyo, Japan) with a 5–13 linear array transducer (width 36 mm); same settings (gain) across all subjects, Orthopedic surgeon as the gold standard.	8 GLCM features including energy, entropy, correlation, homogeneity, inertia, cluster shade, cluster prominence, Haralick correlation (d = 1, θ = 0, 45, 90, 135)	Computer-aided diagnosis based on texture features can differentiate pathological conditions of shoulder muscles. CAD has an accuracy of 87.9% to 88.4% for inflammation, 83.3% for calcific tendinitis, and 92.3% for tears. Cohen’s k was 0.798.
da Silva Pereira Junior et al. [16]	30 men in 2 days, reliability study (biceps brachialis and gastrocnemius lateralis, two planes)	GE LOQIQ E, GE Healthcare, linear array, 40 mm width, 8 MHz, depth 70 mm, RO1 20 × 20 pixels	5 complexity curve-based features: maximum value of transitions, averaged value of transitions, sample mean of the gray-level value weighted by the number of transitions, sample standard deviation of the gray-level value weighted by the number of transitions, and entropy.	The variability of the parameters was slimmer among the subjects for both probe orientations and on 2 days.
Martínez-Payá, et al. [17]	26 amyotrophic lateral sclerosis, 26 controls (biceps brachialis (BB), Forearm flexors (FF), quadriceps femoris (QF), tibialis anterior (TA), bilateral transverse)	LOGIQ BT12, GE Healthcare, 5–13 MHz linear array transducer, depth 5 cm for tibialis anterior and 6 cm for others, same settings for all subjects, 820 × 614 pixels, TIFF (ROI 71 × 40 TA, 73 × 73 others)	Echointensity, echovariation, GLCM (correlation, contrast, energy, entropy, homogeneity)	The combined echovariation and GLCM improves AUC of the logistic regression model. GLCM has AUC 0.849/0.874/0.977/0.934, sensitivity 0.81/0.79/0.94/0.85, and specificity 0.73/0.83/0.94/0.88 for BB/FF/QF/TA.
Sogawa, et al. [18]	25 patients (18 males and 7 females) with neurogenic diseases, 23 with myogenic diseases, and 21 controls (12 males and 9 females), (medial gastrocnemius)	LOGIQ 7, GE Healthcare, 11 MHz linear array transducer, eclipse ROI; assessor blinded	Histogram-derived texture, run-length matrix, autoregressive model, GLCM with linear discriminant analysis, quadratic discriminant analysis, sequential minimal estimation, k-NN, SVM, random forest	Texture analysis can enable differentiation between neurogenic and myogenic diseases. AUCs were 0.70–0.90 between control and neurogenic patients, 0.57–0.80 between control and myogenic patients, and 0.95–1.0 between neurogenic and myogenic patients.
Watanabe, et al. [19]	145 participants in 6 groups based on age and gender: younger group (<44 years, *n* = 68), middle-aged (45–64 years, *n* = 31), the elderly (≥65, *n* = 46) (quadriceps femoris)	LOGIQ E, GE Healthcare, 6–14 MHz	Mean, skewness, kurtosis, inverse difference moment, sum of entropy, angular second moment/energy	Skewness, kurtosis, inverse difference moment, angular second moment and muscle strength significantly decreased in elderly people and mean and sum of entropy significantly decreased in young people.
Dubois, et al. [20]	26 patients (15 males and 11 females) with sporadic inclusion body myositis for gain 40, 50, 60, 70%. 10 Duchenne muscular dystrophy and 10 of 26 patients measured twice for between-day reliability; 16 healthy controls (short head of biceps brachii)	Aixplorer ultrasound device (V9.2, Supersonic Imagine, Aix-en-Provence, France), 4–15 MHz linear array, DICOM, ROI 0.25, 1, 4, 9, 16, 25 and 36 mm^2^	Mean muscle echo intensity (gray-scale index), texture anisotropy index (TAI, higher order feature)	TAI was less sensitive to gain settings that gray-scale index. TAI is correlated with elbow flexor strength. TAI values were less influenced by gain settings than gray-scale index. Patients with muscular dystrophy had lower TAI than healthy controls (*p* < 0.05).
Kumbhare, et al. [21]	18 patients with active myofascial pain, 19 patients with latent myofascial pain, 24 healthy controls (trapezius)	Ultrasonix ultrasound system (BK Medical Systems, BC, Canada), 15 MHz, depth at 3 cm, same settings, ROI 150 × 80 pixels (61 subjects × 30 frames/s × 10 s)	Haralick (contrast, correlation, energy, homogeneity), Galloway (SRE, LRE, RLN, RP, GLN, LGRE, HGRE), and Blob analysis, local binary pattern analysis	Ultrasound texture analysis was sensitive in diagnosing active and latent myofascial and healthy controls. The first 8 features explained 94.92% of the ultrasound image characteristics.
Matta, et al. [22]	13 healthy women for eccentric exercise-induced muscle damage (brachialis, transverse)	Nemio XG ultrasound, Toshiba, Japan, 7.5 MHz, width 38 mm, ROI 25 × 25 pixels	Echo intensity, contrast, correlation	The increased texture correlation represents high similarity between gray levels.
Nodera, et al. [23]	42 healthy individuals aged between 21 and 88 years (right medial gastrocnemius)	LOGIQ7 (GE Healthcare), 11 MHz linear array transducer, bitmap format, eclipse-shape ROI 23,000 to 92,000 pixels	283 texture parameters, gradient, run length matrix, co-occurrence matrix, autoregressive and wavelets, theta 4; delta 1, 2, 3, 4, 5 pixels	Texture features are age dependent. Significanat increases were observed for correlation at 48, 72, and 96 h and echointensity at 72 and 96 h after eccentric contraction. The features related to run-length matrix and autoregressive model could have clinical applications.
Behr, et al. [24]	21 patients with active myofascial pain and 19 patients with latent myofascial pain syndrome and 29 controls (upper trapezius)	X-porte US system (SonoSite, Ontario, Canada), 5–15 MHz, depth 2.7 cm, same settings. ROI 167 × 83 pixel (1.0 × 0.5 cm) for a total of 917 images	76 GLCM and 7 GLRL, 5 first-order features with SVM	SVM classifier based on 88 texture features can differentiate myofascial pain from healthy controls. Machine learning model: 86.96% accuracy, a Matthews correlation coefficient of 0.724, 88% sensitivity, and 86% specificity. Ultrasound textrue analysis is feasible for the classification of healthy and myofascial pain syndrome muscles using a binary SVM classifier.
Chang, et al. [25]	191 shoulder images including 89 supraspinatus tendinopathy and 102 supraspinatus tear from 136 patients (61 males and 75 females) (supraspinatus)	Aloka (Hitachi) with 5–13 MHz (8 MHz used for 4 cm depth)	Histogram (mean, variance, skewness, kurtosis), 14 texture based on 8 × 8 GLCM: autocorrelation contrast, correlation, cluster prominence, cluster shade, dissimilarity, energy, entropy, homogeneity, difference variance, difference entropy, information measure of correlation, inverse difference normalized, inverse difference moment	The proposed system based on texture and intensity can diagnose the supraspinatus tear. The system has an accuracy rate of 92% (176/191) and an area under receiver operating characteristic curve of 0.9694.
Katakis, et al. [26]	74 young individuals (40 males and 34 females) for the gender effects on muscles Biceps (longitudinal), tibialis anterior, long and trans) gastrocnemius medialis (long and trans), and rectus femoris (long and trans)	LOGIQ P9, GE Healthcare, depth at 10 Mhz, gain 50%, dynamic range at 66 DB, depth at 4 cm, except rectus femoris at 6 cm	Scale invariant feature transform with SVM	Machine learning (SVM) with texture analysis can classify gender and muscle types. The accuracy ranged from 69.5–94.52%.
Nodera, et al. [27]	11 patients with inclusion body myositis (IBM), 19 patients with myotonic dystrophy (MD), 21 patients with polymyositis- dermatomyositis (PM) (medial gastrocnemius)	LOGIQ 7 (GE Healthcare), 11 MHz linear array transducer3 circular-shaped ROI for a total of 4 cm^2^.	LIFEx program (IMIV, CEA, France), 40 texture features including GLCM, neighborhood gray-level different matrix, gray-level run length matrix, the gray-level zone length, machine learning	Three-group analysis achieved up to 58.8% accuracy, and two-group analysis of IBM plus PM-MD versus MD showed 78.4% accuracy. Despite a small number of subjects, texture analysis of msucle ultrasound with machine learning was useful in identifying myopathic conditions.
Ríos-Díaz, et al. [28]	59 patients with amyotrophic lateral sclerosis (35 males and 24 females) and 20 healthy controls (10 males and 10 females) (abductor pollicis brevis, bilateral transverse)	Canon Medical Systems Aplio XG, 7–13 MHz, gain 80 dB, depth at 13 MHz	Echointensity, echovariation and GLCM (energy, contrast, correlation, homogeneity, entropy)	Combining ultrasound with biomarkers provides better accuracy for monitoring ALS progression (AUC 82%, sensitivity 87%, specificity 42%). The study indicates that both biomarkers and ultrasound texture analysis should be combined to increase the accuracy.
Behr, et al. [29]	57 patients with fibromyalgia (fibro score 20.8) and 51 healthy controls (fibro score 5.42) (trapezius)	X-porte ultrasound system (SonoSite Canada, Toronto, Canada, depth 3.0 cm, 6–15 MHz linear array, ROI 250 × 115 pixels	31 textures (19 GLCM, 7 GLRM, 5 first-order) combined with support vector machine and logistic regression	The accuracy of SVM and logistic regression models was computed to be 84.1% and 66%, respectively. SVM of ultrasound textrue features demonstrated clinically relevant performance levels.
Kumbhare, et al. [30]	17 patients (5 males and 12 females) with myofascial pain syndrome and 15 controls (8 males and 7 females) (trapezius)	Ultrasonix ultrasound system (Sonixtouch Q+, BK Ultrasound, Canada, 5–15 MHz, depth 2 cm, setting the same) rectangular RO1	Blob area, count and local binary patterns (LBP) with PCA and MANOVA classification	Texture analysis can differentiate between healthy and myofascial pain. A combination of LBP and blob explains 92.55% of the cumulative variance.
Li, et al. [31]	12 healthy individuals performed isometric contractions at 20, 30, 40, 50% MVC (biceps)	M5/M5T B-mode imaging system (Mindray Medical International Limited, Shenzhen, China), 7.5 MhZ, 4 cm depth, setting constant rectangular ROI	Ultrasound image 2D entropy	The decline slope of ultrasound image entropy was basically the same at different contraction intensities. Ultrasound image entropy could be used to assess muscle fatigue.
Paris, et al. [32]	32 healthy adults (16 males and 16 females), ultrasound image resolution/depth (rectus femoris, transverse)	M-Turbo, SonoSite, 5–10 MHz, DICOM at depth of 9.0, 7.3, 5.9 and 4.7 cm,	Echonenicity (mean intensity, kurtosis, energy), (energy, correlation, contrast, 1–10 pixels, 0, 45, 90, 135°), (local binary patterns, circular radius of 5 and 8 sampling points)	Ultrasound image resolution influences texture analysis results. Image resolution should be fixed within and between individuals when evalauting msucle composition using ultrasound.
Sancar, et al. [33]	63 females with myofascial pain syndrome and 20 female healthy controls (trapezius)	Logiq 5 pro machine, 6–18 MHz linear array transducer, depth 5 cm, settings kept the same manual segmentation, between 580 × 125 and 540 × 110 pixels	Blob texture, 0 darkest pixel and 255 brightest pixel, echointensity	The decreased blob size corresponds to a decrease in pain and disability level. Ultrasound texture feature of blob size and count changes correspond to rountine clinical outcomes after physical therapy.
Wilkinson, et al. [34]	90 patients (40 males and 50 females) with chronic kidney disease (rectus femoris)	7.5 MHz, 58 dB gain linear array transducer transverse plan, ROI 50 × 50 pixels	Echointensity, GLCM-based analysis: energy, entropy, homogeneity, correlation and contrast	All GLCM parameters were associated with msucle function although the largest association was seen with image entropy. Texture analysis may provide a novel indicator of muscle quality that is robust to changes in scanner settings.
Bell, et al. [35]	18 people (15 males and 3 females) with type 2 diabetes and prediabetes, 18 controls (15 males and 3 females) (rectus abdominis, rectus femoris, transverse)	5–10 MHz linear array transducer, 3 ROIs at different depths (7.3, 5.9, 4.7 cm)	Histogram kurtosis, GLCM energy, contrast, correlation and homogeneity. Local binary pattern and blob analysis	Ultrasound-based texture features correspond with the muscle strength of rectus abdominisa (histogram skew) and insulin resistance of rectus femoris (LBP entropy). The is the first study to report that ultrasound texure features correspond with functional outcomes.
Escriche-Escuder, et al. [36]	13 women with metastic breast cancer, 12-week exercise intervention (30 min strength and 20 min aerobic exercise) (quadriceps, biceps and brachialis)	12 MHz, 70% gain, the depth was adjusted for each participant, 800 × 652 pixels, 96 dpi (1 cm in horizontal length)	Contrast, correlation, energy, homogeneity, entropy	Contrast, homogeneity, and entropy were correlated with changes after exercise intervention.
Mirón Mombiela and Borrás [37]	101 older people (55 males) at risk for frailty (rectus femoris and vastus lateralis)	LOGIC S7 (GE Healthcare) with 6–15 MHz linear transducer	3 histogram, 9 GLCM, 13 GLRL, 13 gray-level size zone matrix, 5 neighborhood gray-tone difference matrix at 1-pixel distance in 0, 45, 90, 135° with machine learning models	The study showed that the heterogeneity of muscle was associated with an increased incidence of hearing impairment, stroke, myocardial infraction, dementia, and falls. The msucle radiomic model needs to be validated. Texture features can predict the frailty of a phenotype.
Tang, et al. [38]	115 adults with sarcopenia (79 older adults and 36 young adults (younger than 60 years)) (rectus femoris)	Multi-frequency linear array, 67 Db, depth at 5 cm 80 × 80 pixels	Local binary pattern (LBP) and GLCM (contrast, correlation, entropy, homogeneity)	LBP combined with GLCM can be used to extract muscle texture features. The constrast, entropy, and homogeneity of the rectus femoris of the older adults with sarcopenia were significantly different from those of the young adults.
Koh, et al. [39]	201 patients with myofascial pain syndrome (trigger point) (trapezius)	SonixTouch 1+, Ultrasonix Medical Corp, Richmond, BC, Canada, depth 2.5 cm, 6–15 MHz linear array, setting the same rectangular ROI	5 first-order (mean, SD, variance, skewness, kurtosis), 19 GLCM at 0, 45, 90, 135º, distance 9 pixels, 7 GLRM, 59 LBP, 3 blob analysis with CNN	Texture analysis with F1score of 0.7135 (sensitivity 0.6821, specificity 0.7823) and CNN with 0.7299 (sensitivity 0.6005, specificity 0.8566) on differentiating MTP.
Mirón-Mombiela, et al. [40]	*Secondary analysis* of [37], 101 frailty (55 males and 46 females) phenotype (rectus femoris and vastus lateralis)	LOGIC S7 (GE Healthcare) with 6–15 MHz linear transducer	43 features: 9 GLCM, 13 GLRM, 13 GLSZM, 5 NGTDM with Naïve Bayes, KNN, multilayer perceptron, random forest, and SVM	Texture can be useful to identify frailty and assess risk prediction with AUC 0.67–0.79. Models developed achieved AUC (0.67–0.79) for caterogrizging frailty. The stepwise multiple logistic regression analysis has 70–87%.
Sahinis and Kellis [41]	10 young males (hamstring)	Logiq E GE Healthcare, 5–13 MhZ, 4 cm field of view, setting constant, depth at 60 mm, gain 59 dB, dynamic range 65 dB, ROI 35 × 35 pixels	Contrast, correlation, entropy, angular second moment, inverse difference moment,	Significant differences among hamstring and measurement locations. Only the correlation feature at the proximal measurement site exhibited a significant relationship with strength.The study indicated significant diffrences among hamstring and measurement locations with respect to texture analysis.
Zadeh, et al. [42]	63 patients with myofascial pain syndrome (30 active, 30 latent) and 30 healthy controls (trapezius)	SonixTouch Q+, Ultrasonix Medical, Richmond, Canada, 6–15 MHz linear array, depth 2.5 cm, same settings, 90 images	LBP, Gabor, and SEGL based on entropy, contrast, correlation, homogeneity, energy, mean, variance, GLCM (8 directions, 1 pixel) and LBP with k-NN, decision tree, random forest, naïve bayes, SVM, ANN	Significant results were seen in almost all ultrasound texture features. The accuracy ranged around 40–55%. Machine learning showed that two texture features, correlation and mean, within the combination of texture features were most important in classifying the 3 groups.
Cruz-Montecinos, et al. [43]	44 healthy (22 males and 22 females) children, gender effects (rectus femoris)	Nemio XG ultrasound system (Toshiba, Japan), 9 MHz linear array, width 38 mm, depth 70 mm, ROI two-thirds of the visible thickness and width of the muscle	Echo intensity, GLCM (homogeneity and correlation)	The study revealed sex-specific differences in echo intensity and homogeneity texture. The study suports the use of ultrasound texture analysis for examing muscle quality in healthy children and those with medical conditions.
Hung and Jan [44]	12 individuals (5 males) for cupping therapy (−225 and −300 mmHg for 5 and 10 min) (triceps)	ProSound A7 (Hitachi Healthcare) with 17–22 MHz linear array, depth at 17 MHz, settings kept the same, DICOM, ROI 90% of the ultrasound image of triceps	GLCM: contrast, correlation, energy, homogeneity	Cupping therapy increases contrast texture and decreases correlation texture of the superficial layer of the triceps.
Jo and Kim [45]	14 healthy young males performing eccentric exercise, immediately post-exercise and after 24, 48, 72, 96 h (long head of biceps brachii)	S12, SonoScape Co, Shenzhen, China, 4–16 MHz linear array, width 50 mm, gain 59 dB, depth 5 cm, focus 2 cm, same settings, ROI 96 × 96 pixels	EI, GLCM (contrast, entropy, energy, homogeneity)	The study demonstrated that EIMD severity is different between LHF and SHB. GLCM is better than echo intensity.
McCrady, et al. [46]	6 patients (4 males) with spinal muscular atrophy, 14 patients (6 amulatory (5 males) and 8 non-ambulatory (8 males)) with Duchenne muscular dystrophy, 16 controls (11 males) (biceps, brachioradialis, extensor carpi radialis, longitudinal, and transverse)	Telemed Medical Systems, Milano, Italy, Depth 50 MM, frequency 8 MHz, gain 88%	Texture anisotropy index, echogenicity	Significant differences were observed in muscle size, shape, and quality with increased disease severity comapred to healthy controls. A multivariable approach combing muscle size and quality improves strength prediction (R^2^ = 0.65).
Mongold, et al. [47]	32 young (18–35 years, 17 males and 15 females), 34 old (65–85 years, 16 males and 18 females) individuals (biceps brachii and vastus lateralis, transverse plan)	Vscan Air (GE Healthcare), 3–12 MHz, depth 5 cm, gain 40%	Echo intensity, homogeneity, 0, 90, 180, 270, angular second moment, contrast, correlation, inverse different moment, and entropy	The study indicates that texture-based parameters provide a robust alternative to echo intensity for assessing muscle composition.
Wilkinson, et al. [48]	*Secondary analysis* of ExTra-CKD TRIAL: 14 patients (7 males) with chronic kidney disease (quadriceps and hamstring, transverse plan)	B-mode 2D ultrasonography (Hitachi EUB-6500) with 7.5 MHz (3 Tesla MRI as a reference standard)	EI, GLCM (angular second moment, entropy, inverse difference moment, correlation, and contrast)	Texture analysis may provide reliable measure of muscle quality. A higher energy with greater muscle percent (β = 0.57), a higher homogeneity with greater muscle percent (β = 0.61).

## Supplementary Materials

The following supporting information can be downloaded at: https://www.mdpi.com/article/10.3390/diagnostics15050524/s1. The PRISMA checklist, the list of excluded studies and reasons, and the detail of quality assessment using QUADAS-2 are provided in the supplemental files.
