# Supplementary material for: Texture Analysis in Musculoskeletal Ultrasonography: A Systematic Review
Source: Diagnostics (Basel). 2025 Feb 21;15(5):524. doi: 10.3390/diagnostics15050524 (PMC11899606; doi:10.3390/diagnostics15050524)
Supplement: Supplementary file 1 [file diagnostics-15-00524-s001.zip › diagnostics-3455222-supplementary.pdf]

PRISMA-DTA Checklist

Section/topic	#	PRISMA-DTA Checklist Item	Reported on page #
**TITLE / ABSTRACT**			
Title	1	Identify the report as a systematic review (+/- meta-analysis) of diagnostic test accuracy (DTA) studies.	Ln 1
Abstract	2	Abstract: See PRISMA-DTA for abstracts.	Ln 7
**INTRODUCTION**			
Rationale	3	Describe the rationale for the review in the context of what is already known.	Ln 58-75
Clinical role of index test	D1	State the scientific and clinical background, including the intended use and clinical role of the index test, and if applicable, the rationale for minimally acceptable test accuracy (or minimum difference in accuracy for comparative design).	Ln 58-67
Objectives	4	Provide an explicit statement of question(s) being addressed in terms of participants, index test(s), and target condition(s).	Ln 71-75
**METHODS**			
Protocol and registration	5	Indicate if a review protocol exists, if and where it can be accessed (e.g., Web address), and, if available, provide registration information including registration number.	Ln 78-81
Eligibility criteria	6	Specify study characteristics (participants, setting, index test(s), reference standard(s), target condition(s), and study design) and report characteristics (e.g., years considered, language, publication status) used as criteria for eligibility, giving rationale.	Ln 94-113
Information sources	7	Describe all information sources (e.g., databases with dates of coverage, contact with study authors to identify additional studies) in the search and date last searched.	Ln 88-90
Search	8	Present full search strategies for all electronic databases and other sources searched, including any limits used, such that they could be repeated.	Ln 90-92
Study selection	9	State the process for selecting studies (i.e., screening, eligibility, included in systematic review, and, if applicable, included in the meta-analysis).	Ln 115-121
Data collection process	10	Describe method of data extraction from reports (e.g., piloted forms, independently, in duplicate) and any processes for obtaining and confirming data from investigators.	Ln 121-124
Definitions for data extraction	11	Provide definitions used in data extraction and classifications of target condition(s), index test(s), reference standard(s) and other characteristics (e.g. study design, clinical setting).	Ln 81-85, 110-113
Risk of bias and applicability	12	Describe methods used for assessing risk of bias in individual studies and concerns regarding the applicability to the review question.	Ln 126-136
Diagnostic accuracy measures	13	State the principal diagnostic accuracy measure(s) reported (e.g. sensitivity, specificity) and state the unit of assessment (e.g. per-patient, per-lesion).	Ln 139-144
Synthesis of results	14	Describe methods of handling data, combining results of studies and describing variability between studies. This could include, but is not limited to: a) handling of multiple definitions of target condition. b) handling of multiple thresholds of test positivity, c) handling multiple index test readers, d) handling of indeterminate test results, e) grouping and comparing tests, f) handling of different reference standards	Ln 144-149

PRISMA-DTA Checklist

Section/topic	#	PRISMA-DTA Checklist Item	Reported on page #
Meta-analysis	D2	Report the statistical methods used for meta-analyses, if performed.	NA
Additional analyses	16	Describe methods of additional analyses (e.g., sensitivity or subgroup analyses, meta-regression), if done, indicating which were pre-specified.	NA
**RESULTS**			
Study selection	17	Provide numbers of studies screened, assessed for eligibility, included in the review (and included in meta-analysis, if applicable) with reasons for exclusions at each stage, ideally with a flow diagram.	Ln 152-156
Study characteristics	18	For each included study provide citations and present key characteristics including: a) participant characteristics (presentation, prior testing), b) clinical setting, c) study design, d) target condition definition, e) index test, f) reference standard, g) sample size, h) funding sources	Ln 158-230
Risk of bias and applicability	19	Present evaluation of risk of bias and concerns regarding applicability for each study.	Ln 232-241
Results of individual studies	20	For each analysis in each study (e.g. unique combination of index test, reference standard, and positivity threshold) report 2x2 data (TP, FP, FN, TN) with estimates of diagnostic accuracy and confidence intervals, ideally with a forest or receiver operator characteristic (ROC) plot.	Ln 166-197
Synthesis of results	21	Describe test accuracy, including variability; if meta-analysis was done, include results and confidence intervals.	Ln 199-230
Additional analysis	23	Give results of additional analyses, if done (e.g., sensitivity or subgroup analyses, meta-regression; analysis of index test: failure rates, proportion of inconclusive results, adverse events).	NA
**DISCUSSION**			
Summary of evidence	24	Summarize the main findings including the strength of evidence.	Ln 244-287
Limitations	25	Discuss limitations from included studies (e.g. risk of bias and concerns regarding applicability) and from the review process (e.g. incomplete retrieval of identified research).	Ln 396-409
Conclusions	26	Provide a general interpretation of the results in the context of other evidence. Discuss implications for future research and clinical practice (e.g. the intended use and clinical role of the index test).	Ln 423-439
**FUNDING**			
Funding	27	For the systematic review, describe the sources of funding and other support and the role of the funders.	NA

Adapted From: McInnes MDF, Moher D, Thombs BD, McGrath TA, Bossuyt PM, The PRISMA-DTA Group (2018). Preferred Reporting Items for a Systematic Review and Meta-analysis of Diagnostic Test Accuracy Studies: The PRISMA-DTA Statement. JAMA. 2018 Jan 23;319(4):388-396. doi: 10.1001/jama.2017.19163.

List of excluded studies after discussions

Eisele, Schmid [1]	Echo intensity only
Cheng, Metreweli [2]	Multiple ultrasound devices
Alvarenga, Teixeira [3]	Used phantom
Zhou, Wei [4]	Skeletal muscle is not the primary research topic; used to segment breast and muscle tissues
Tsui, Huang [5]	In vitro vocal tissues
Qiu, Zhao [6]	No full text
Pazinato, Stein [7]	Cardiac muscles
Cunningham, Harding [8]	Texture analysis is part of another imaging modality's protocol
Hu, Fu [9]	Echo intensity only
Gabison, Mathur [10]	Multiple ultrasound devices
Sikdar, Diao [11]	Radiofrequency ultrasound
Chiou, Yeh [12]	Multiple ultrasound devices
Gabison, Mathur [13]	Multiple ultrasound devices
Evans, Behr [14]	Protocol only
De-la-Cruz-Torres, Navarro-Flores [15]	Tendon research
Liao, Wang [16]	Used to reconstruct ultrasound images; not the primary research question
Wu, Barrere [17]	1 patient and 1 control subject

1. Eisele, R., et al., *Soft tissue texture analysis by B-mode-ultrasound in the evaluation of impairment in chronic low back pain.* Eur J Ultrasound, 1998. **8**(3): p. 167-75.
2. Cheng, J.C., et al., *Correlation of ultrasonographic imaging of congenital muscular torticollis with clinical assessment in infants.* Ultrasound Med Biol, 2000. **26**(8): p. 1237-41.
3. Alvarenga, A.V., et al., *Influence of temperature variations on the entropy and correlation of the Grey-Level Co-occurrence Matrix from B-Mode images.* Ultrasonics, 2010. **50**(2): p. 290-3.
4. Zhou, C., et al., *Computerized image analysis: texture-field orientation method for pectoral muscle identification on MLO-view mammograms.* Med Phys, 2010. **37**(5): p. 2289-99.
5. Tsui, P.H., et al., *Characterization of lamina propria and vocal muscle in human vocal fold tissue by ultrasound Nakagami imaging.* Med Phys, 2011. **38**(4): p. 2019-26.
6. Qiu, S., et al., *Ultrasound Image Analysis on Muscle Texture of Vastus Intermedius and Rectus Femoris Under Neuromuscular Electrical Stimulation.* Journal of Medical Imaging and Health Informatics, 2015. **5**(2): p. 342-349.
7. Pazinato, D.V., et al., *Pixel-Level Tissue Classification for Ultrasound Images.* IEEE J Biomed Health Inform, 2016. **20**(1): p. 256-67.
8. Cunningham, R.J., P.J. Harding, and I.D. Loram, *Real-Time Ultrasound Segmentation, Analysis and Visualisation of Deep Cervical Muscle Structure.* IEEE Trans Med Imaging, 2017. **36**(2): p. 653-665.
9. Hu, C.F., et al., *Longitudinal follow-up of muscle echotexture in infants with congenital muscular torticollis.* Medicine (Baltimore), 2017. **96**(6): p. e6068.

10. Gabison, S., et al., *Quantitative ultrasound imaging over the ischial tuberosity: An exploratory study to inform tissue health.* J Tissue Viability, 2018. **27**(3): p. 173-180.
11. Sikdar, S., et al., *Quantification of Muscle Tissue Properties by Modeling the Statistics of Ultrasound Image Intensities Using a Mixture of Gamma Distributions in Children With and Without Cerebral Palsy.* J Ultrasound Med, 2018. **37**(9): p. 2157-2169.
12. Chiou, H.J., et al., *Efficacy of Quantitative Muscle Ultrasound Using Texture-Feature Parametric Imaging in Detecting Pompe Disease in Children.* Entropy (Basel), 2019. **21**(7).
13. Gabison, S., et al., *The relationship between pressure offloading and ischial tissue health in individuals with spinal cord injury: An exploratory study.* J Spinal Cord Med, 2019. **42**(sup1): p. 186-195.
14. Evans, V., et al., *Quantitative response of healthy muscle following the induction of capsaicin: an exploratory randomized controlled trial.* Trials, 2020. **21**(1): p. 1020.
15. De-la-Cruz-Torres, B., et al., *Ultrasound Imaging Evaluation of Textural Features in Athletes with Soleus Pathology-A Novel Case-Control Study.* Int J Environ Res Public Health, 2021. **18**(4).
16. Liao, A.H., et al., *Computer-Aided Diagnosis of Duchenne Muscular Dystrophy Based on Texture Pattern Recognition on Ultrasound Images Using Unsupervised Clustering Algorithms and Deep Learning.* Ultrasound Med Biol, 2024. **50**(7): p. 1058-1068.
17. Wu, Y., et al., *High-frequency Quantitative Ultrasound Imaging of Human Rotator Cuff Muscles: Assessment of Repeatability and Reproducibility.* Ultrason Imaging, 2024. **46**(1): p. 56-70.

Supplementary file 2: Table. Quality assessment using QUADAS-2

	Bias				Concerns regarding applicability		
Study	Patient selection	index test	Reference standard	Flow and timing	Patient selection	index test	Reference standard
Nielsen, Jensen [1]	☹ high	☹ high	☹ high	⯑ unclear	☺ low	☺ low	☹ high
Turo, Otto [2]	☹ high	☹ high	☹ high	⯑ unclear	☺ low	☺ low	☹ high
König, Steffen [3]	☹ high	☹ high	☹ high	⯑ unclear	☺ low	☺ low	☹ high
Molinari, Caresio [4]	☹ high	☹ high	☹ high	⯑ unclear	☺ low	☺ low	☹ high
Chang, Lee [5]	☹ high	☹ high	☹ high	⯑ unclear	☺ low	☺ low	☹ high
da Silva Pereira Junior, da Matta [6]	☹ high	☹ high	☹ high	⯑ unclear	☺ low	☺ low	☹ high
Martínez-Payá, Ríos-Díaz [7]	☹ high	☹ high	☹ high	⯑ unclear	☺ low	☺ low	☹ high
Sogawa, Nodera [8]	☹ high	☹ high	☹ high	⯑ unclear	☺ low	☺ low	☹ high
Watanabe, Murakami [9]	☹ high	☹ high	☹ high	⯑ unclear	☺ low	☺ low	☹ high
Dubois, Bachasson [10]	☹ high	☹ high	☹ high	⯑ unclear	☺ low	☺ low	☹ high
Kumbhare, Ahmed [11]	☹ high	☹ high	☹ high	⯑ unclear	☺ low	☺ low	☹ high
Matta, Pereira [12]	☹ high	☹ high	☹ high	⯑ unclear	☺ low	☺ low	☹ high
Nodera, Sogawa [13]	☹ high	☹ high	☹ high	⯑ unclear	☺ low	☺ low	☹ high
Behr, Noseworthy [14]	☹ high	☹ high	☹ high	⯑ unclear	☺ low	☺ low	☹ high
Chang, Lee [15]	☹ high	☹ high	☹ high	⯑ unclear	☺ low	☺ low	☹ high
Katakis, Barotsis [16]	☹ high	☹ high	☹ high	⯑ unclear	☺ low	☺ low	☹ high
Nodera, Sogawa [17]	☹ high	☹ high	☹ high	⯑ unclear	☺ low	☺ low	☹ high
Ríos-Díaz, Del Baño-Aledo [18]	☹ high	☹ high	☹ high	⯑ unclear	☺ low	☺ low	☹ high
Behr, Saiel [19]	☹ high	☹ high	☹ high	⯑ unclear	☺ low	☺ low	☹ high
Kumbhare, Shaw [20]	☹ high	☹ high	☹ high	⯑ unclear	☺ low	☺ low	☹ high
Li, Yang [21]	☹ high	☹ high	☹ high	⯑ unclear	☺ low	☺ low	☹ high
Paris, Bell [22]	☹ high	☹ high	☹ high	⯑ unclear	☺ low	☺ low	☹ high
Sancar, Keniş-Coşkun [23]	☹ high	☹ high	☹ high	⯑ unclear	☺ low	☺ low	☹ high
Wilkinson, Ashman [24]	☹ high	☹ high	☹ high	⯑ unclear	☺ low	☺ low	☹ high
Bell, Paris [25]	☹ high	☹ high	☹ high	⯑ unclear	☺ low	☺ low	☹ high
Escriche-Escuder, Trinidad-Fernández [26]	☹ high	☹ high	☹ high	⯑ unclear	☺ low	☺ low	☹ high
Mirón Mombiela and Borrás [27]	☹ high	☹ high	☹ high	⯑ unclear	☺ low	☺ low	☹ high
Tang, Yang [28]	☹ high	☹ high	☹ high	⯑ unclear	☺ low	☺ low	☹ high
Koh, Dilek [29]	☹ high	☹ high	☹ high	⯑ unclear	☺ low	☺ low	☹ high
Mirón-Mombiela, Ruiz-España [30]	☹ high	☹ high	☹ high	⯑ unclear	☺ low	☺ low	☹ high

Sahinis and Kellis [31]	☹ high	☹ high	☹ high	◈ unclear	☺ low	☺ low	☹ high
Zadeh, Koh [32]	☹ high	☹ high	☹ high	◈ unclear	☺ low	☺ low	☹ high
Cruz-Montecinos, Pinto [33]	☹ high	☹ high	☹ high	◈ unclear	☺ low	☺ low	☹ high
Hung and Jan [34]	☹ high	☹ high	☹ high	◈ unclear	☺ low	☺ low	☹ high
Jo and Kim [35]	☹ high	☹ high	☹ high	◈ unclear	☺ low	☺ low	☹ high
McCrady, Masterson [36]	☹ high	☹ high	☹ high	◈ unclear	☺ low	☺ low	☹ high
Mongold, Georgiev [37]	☹ high	☹ high	☹ high	◈ unclear	☺ low	☺ low	☹ high
Wilkinson, Baker [38]	☹ high	☹ high	☺ low	◈ unclear	☺ low	☺ low	☹ high

Supplemental Table. Quality assessment using QUADAS-2

	**Bias**				**Concerns regarding applicability**		
Study	Patient selection	index test	Reference standard	Flow and timing	Patient selection	index test	Reference standard
Nielsen, Jensen et al. (2006)	☹ high	☹ high	☹ high	◈ unclear	☺ low	☺ low	☹ high
Turo, Otto et al. (2013)	☹ high	☹ high	☹ high	◈ unclear	☺ low	☺ low	☹ high
König, Steffen et al. (2015)	☹ high	☹ high	☹ high	◈ unclear	☺ low	☺ low	☹ high
Molinari, Caresio et al. (2015)	☹ high	☹ high	☹ high	◈ unclear	☺ low	☺ low	☹ high
Chang, Lee et al. (2016)	☹ high	☹ high	☹ high	◈ unclear	☺ low	☺ low	☹ high
da Silva Pereira Junior, da Matta et al. (2017)	☹ high	☹ high	☹ high	◈ unclear	☺ low	☺ low	☹ high
Martínez-Payá, Ríos-Díaz et al. (2017)	☹ high	☹ high	☹ high	◈ unclear	☺ low	☺ low	☹ high
Sogawa, Nodera et al. (2017)	☹ high	☹ high	☹ high	◈ unclear	☺ low	☺ low	☹ high
Watanabe, Murakami et al. (2017)	☹ high	☹ high	☹ high	◈ unclear	☺ low	☺ low	☹ high
Dubois, Bachasson et al. (2018)	☹ high	☹ high	☹ high	◈ unclear	☺ low	☺ low	☹ high
Kumbhare, Ahmed et al. (2018)	☹ high	☹ high	☹ high	◈ unclear	☺ low	☺ low	☹ high
Matta, Pereira et al. (2018)	☹ high	☹ high	☹ high	◈ unclear	☺ low	☺ low	☹ high
Nodera, Sogawa et al. (2018)	☹ high	☹ high	☹ high	◈ unclear	☺ low	☺ low	☹ high

Behr, Noseworthy et al. (2019)	☹ high	☹ high	☹ high	⯑ unclear	☺ low	☺ low	☹ high
Chang, Lee et al. (2019)	☹ high	☹ high	☹ high	⯑ unclear	☺ low	☺ low	☹ high
Katakis, Barotsis et al. (2019)	☹ high	☹ high	☹ high	⯑ unclear	☺ low	☺ low	☹ high
Nodera, Sogawa et al. (2019)	☹ high	☹ high	☹ high	⯑ unclear	☺ low	☺ low	☹ high
Ríos-Díaz, Del Baño-Aledo et al. (2019)	☹ high	☹ high	☹ high	⯑ unclear	☺ low	☺ low	☹ high
Behr, Saiel et al. (2020)	☹ high	☹ high	☹ high	⯑ unclear	☺ low	☺ low	☹ high
Kumbhare, Shaw et al. (2020)	☹ high	☹ high	☹ high	⯑ unclear	☺ low	☺ low	☹ high
Li, Yang et al. (2020)	☹ high	☹ high	☹ high	⯑ unclear	☺ low	☺ low	☹ high
Paris, Bell et al. (2020)	☹ high	☹ high	☹ high	⯑ unclear	☺ low	☺ low	☹ high
Sancar, Keniş-Coşkun et al. (2021)	☹ high	☹ high	☹ high	⯑ unclear	☺ low	☺ low	☹ high
Wilkinson, Ashman et al. (2021)	☹ high	☹ high	☹ high	⯑ unclear	☺ low	☺ low	☹ high
Bell, Paris et al. (2022)	☹ high	☹ high	☹ high	⯑ unclear	☺ low	☺ low	☹ high
Escriche-Escuder, Trinidad-Fernández et al. (2022)	☹ high	☹ high	☹ high	⯑ unclear	☺ low	☺ low	☹ high
Mirón Mombiela and Borrás (2022)	☹ high	☹ high	☹ high	⯑ unclear	☺ low	☺ low	☹ high
Tang, Yang et al. (2022)	☹ high	☹ high	☹ high	⯑ unclear	☺ low	☺ low	☹ high
Koh, Dilek et al. (2023)	☹ high	☹ high	☹ high	⯑ unclear	☺ low	☺ low	☹ high
Mirón-Mombiela,	☹ high	☹ high	☹ high	⯑ unclear	☺ low	☺ low	☹ high

Ruiz-España et al. (2023)							
Sahinis and Kellis (2023)	☹ high	☹ high	☹ high	⟐ unclear	☺ low	☺ low	☹ high
Zadeh, Koh et al. (2023)	☹ high	☹ high	☹ high	⟐ unclear	☺ low	☺ low	☹ high
Cruz-Montecinos, Pinto et al. (2024)	☹ high	☹ high	☹ high	⟐ unclear	☺ low	☺ low	☹ high
Hung and Jan (2024)	☹ high	☹ high	☹ high	⟐ unclear	☺ low	☺ low	☹ high
Jo and Kim (2024)	☹ high	☹ high	☹ high	⟐ unclear	☺ low	☺ low	☹ high
McCrady, Masterson et al. (2024)	☹ high	☹ high	☹ high	⟐ unclear	☺ low	☺ low	☹ high
Mongold, Georgiev et al. (2024)	☹ high	☹ high	☹ high	⟐ unclear	☺ low	☺ low	☹ high
Wilkinson, Baker et al. (2024)	☹ high	☹ high	☺ low	⟐ unclear	☺ low	☺ low	☹ high

Behr, M., M. Noseworthy and D. Kumbhare (2019). "Feasibility of a Support Vector Machine Classifier for Myofascial Pain Syndrome: Diagnostic Case-Control Study." J Ultrasound Med **38**(8): 2119-2132.

Behr, M., S. Saiel, V. Evans and D. Kumbhare (2020). "Machine Learning Diagnostic Modeling for Classifying Fibromyalgia Using B-mode Ultrasound Images." Ultrason Imaging **42**(3): 135-147.

Bell, K. E., M. T. Paris, E. Avrutin and M. Mourtzakis (2022). "Altered features of body composition in older adults with type 2 diabetes and prediabetes compared with matched controls." J Cachexia Sarcopenia Muscle **13**(2): 1087-1099.

Chang, R. F., C. C. Lee and C. M. Lo (2016). "Computer-Aided Diagnosis of Different Rotator Cuff Lesions Using Shoulder Musculoskeletal Ultrasound." Ultrasound Med Biol **42**(9): 2315-2322.

Chang, R. F., C. C. Lee and C. M. Lo (2019). "Quantitative diagnosis of rotator cuff tears based on sonographic pattern recognition." PLoS One **14**(2): e0212741.

Cruz-Montecinos, C., M. D. Pinto and R. S. Pinto (2024). "Sex differences in quantitative ultrasonographic measurements of the rectus femoris in children." J Anat.

da Silva Pereira Junior, N., T. T. da Matta, A. V. Alvarenga, W. C. de Albuquerque Pereira and L. F. de Oliveira (2017). "Reliability of ultrasound texture measures of Biceps Brachialis and Gastrocnemius Lateralis muscles' images." Clin Physiol Funct Imaging **37**(1): 84-88.

Dubois, G. J. R., D. Bachasson, L. Lacourpaille, O. Benveniste and J. Y. Hogrel (2018). "Local Texture Anisotropy as an Estimate of Muscle Quality in Ultrasound Imaging." Ultrasound Med Biol **44**(5): 1133-1140.

Escriche-Escuder, A., M. Trinidad-Fernández, B. Pajares, M. Iglesias-Campos, E. Alba, J. M. García-Almeida, C. Roldán-Jiménez and A. I. Cuesta-Vargas (2022). "Responsiveness of the new index muscular

echotexture in women with metastatic breast cancer: an exercise intervention study." Sci Rep **12**(1): 15148.
Hung, I. Y. and Y. K. Jan (2024). "Using texture analysis of ultrasound images to assess the effect of cupping therapy on muscle quality of the triceps." PLoS One **19**(3): e0301221.
Jo, H. D. and M. K. Kim (2024). "Identification of EIMD Level Differences Between Long- and Short Head of Biceps Brachii Using Echo Intensity and GLCM Texture Features." Res Q Exerc Sport **95**(2): 441-449.
Katakis, S., N. Barotsis, D. Kastaniotis, C. Theoharatos, P. Tsiganos, G. Economou, E. Panagiotopoulos, S. Fotopoulos and G. Panayiotakis (2019). "Muscle Type and Gender Recognition Utilising High-Level Textural Representation in Musculoskeletal Ultrasonography." Ultrasound Med Biol **45**(7): 1562-1573.
Koh, R. G. L., B. Dilek, G. Ye, A. Selver and D. Kumbhare (2023). "Myofascial Trigger Point Identification in B-Mode Ultrasound: Texture Analysis Versus a Convolutional Neural Network Approach." Ultrasound Med Biol **49**(10): 2273-2282.
König, T., J. Steffen, M. Rak, G. Neumann, L. von Rohden and K. D. Tönnies (2015). "Ultrasound texture-based CAD system for detecting neuromuscular diseases." Int J Comput Assist Radiol Surg **10**(9): 1493-1503.
Kumbhare, D., S. Shaw, S. Ahmed and M. D. Noseworthy (2020). "Quantitative ultrasound of trapezius muscle involvement in myofascial pain: comparison of clinical and healthy population using texture analysis." J Ultrasound **23**(1): 23-30.
Kumbhare, D. A., S. Ahmed, M. G. Behr and M. D. Noseworthy (2018). "Quantitative Ultrasound Using Texture Analysis of Myofascial Pain Syndrome in the Trapezius." Crit Rev Biomed Eng **46**(1): 1-31.
Li, P., X. Yang, G. Yin and J. Guo (2020). "Skeletal Muscle Fatigue State Evaluation with Ultrasound Image Entropy." Ultrason Imaging **42**(6): 235-244.
Martínez-Payá, J. J., J. Ríos-Díaz, M. E. Del Baño-Aledo, J. I. Tembl-Ferrairó, J. F. Vazquez-Costa and F. Medina-Mirapeix (2017). "Quantitative Muscle Ultrasonography Using Textural Analysis in Amyotrophic Lateral Sclerosis." Ultrason Imaging **39**(6): 357-368.
Matta, T. T. D., W. C. A. Pereira, R. Radaelli, R. S. Pinto and L. F. Oliveira (2018). "Texture analysis of ultrasound images is a sensitive method to follow-up muscle damage induced by eccentric exercise." Clin Physiol Funct Imaging **38**(3): 477-482.
McCrady, A. N., C. D. Masterson, L. E. Barnes, R. J. Scharf and S. S. Blemker (2024). "Development of an ultrasound-based metric of muscle functional capacity for use in patients with neuromuscular disease." Muscle Nerve **70**(6): 1205-1214.
Mirón-Mombiela, R., S. Ruiz-España, D. Moratal and C. Borrás (2023). "Assessment and risk prediction of frailty using texture-based muscle ultrasound image analysis and machine learning techniques." Mech Ageing Dev **215**: 111860.
Mirón Mombiela, R. and C. Borrás (2022). "The Usefulness of Radiomics Methodology for Developing Descriptive and Prognostic Image-Based Phenotyping in the Aging Population: Results From a Small Feasibility Study." Front Aging **3**: 853671.
Molinari, F., C. Caresio, U. R. Acharya, M. R. Mookiah and M. A. Minetto (2015). "Advances in quantitative muscle ultrasonography using texture analysis of ultrasound images." Ultrasound Med Biol **41**(9): 2520-2532.
Mongold, S. J., C. Georgiev, G. Naeije, M. Vander Ghinst, M. S. Stock and M. Bourguignon (2024). "Age-related changes in ultrasound-assessed muscle composition and postural stability." Sci Rep **14**(1): 18688.
Nielsen, P. K., B. R. Jensen, T. Darvann, K. Jørgensen and M. Bakke (2006). "Quantitative ultrasound tissue characterization in shoulder and thigh muscles--a new approach." BMC Musculoskelet Disord **7**: 2.
Nodera, H., K. Sogawa, N. Takamatsu, S. Hashiguchi, M. Saito, A. Mori, Y. Osaki, Y. Izumi and R. Kaji (2019). "Texture analysis of sonographic muscle images can distinguish myopathic conditions." J Med Invest **66**(3.4): 237-247.

Nodera, H., K. Sogawa, N. Takamatsu, A. Mori, H. Yamazaki, Y. Izumi and R. Kaji (2018). "Age-dependent texture features in skeletal muscle ultrasonography." J Med Invest **65**(3.4): 274-279.
Paris, M. T., K. E. Bell, E. Avrutin and M. Mourtzakis (2020). "Ultrasound image resolution influences analysis of skeletal muscle composition." Clin Physiol Funct Imaging **40**(4): 277-283.
Ríos-Díaz, J., M. E. Del Baño-Aledo, J. I. Tembl-Ferrairó, M. J. Chumillas, J. F. Vázquez-Costa and J. J. Martínez-Payá (2019). "Quantitative neuromuscular ultrasound analysis as biomarkers in amyotrophic lateral sclerosis." Eur Radiol **29**(8): 4266-4275.
Sahinis, C. and E. Kellis (2023). "Hamstring Muscle Quality Properties Using Texture Analysis of Ultrasound Images." Ultrasound Med Biol **49**(2): 431-440.
Sancar, M., Ö. Keniş-Coşkun, O. H. Gündüz and D. Kumbhare (2021). "Quantitative Ultrasound Texture Feature Changes With Conservative Treatment of the Trapezius Muscle in Female Patients With Myofascial Pain Syndrome." Am J Phys Med Rehabil **100**(11): 1054-1061.
Sogawa, K., H. Nodera, N. Takamatsu, A. Mori, H. Yamazaki, Y. Shimatani, Y. Izumi and R. Kaji (2017). "Neurogenic and Myogenic Diseases: Quantitative Texture Analysis of Muscle US Data for Differentiation." Radiology **283**(2): 492-498.
Tang, X., Y. Yang, L. Huang and L. Qiu (2022). "The Application of Texture Feature Analysis of Rectus Femoris Based on Local Binary Pattern (LBP) Combined With Gray-Level Co-Occurrence Matrix (GLCM) in Sarcopenia." J Ultrasound Med **41**(9): 2169-2179.
Turo, D., P. Otto, J. P. Shah, J. Heimur, T. Gebreab, M. Zaazhoa, K. Armstrong, L. H. Gerber and S. Sikdar (2013). "Ultrasonic characterization of the upper trapezius muscle in patients with chronic neck pain." Ultrason Imaging **35**(2): 173-187.
Watanabe, T., H. Murakami, D. Fukuoka, N. Terabayashi, S. Shin, T. Yabumoto, H. Ito, H. Fujita, T. Matsuoka and M. Seishima (2017). "Quantitative Sonographic Assessment of the Quadriceps Femoris Muscle in Healthy Japanese Adults." J Ultrasound Med **36**(7): 1383-1395.
Wilkinson, T. J., J. Ashman, L. A. Baker, E. L. Watson and A. C. Smith (2021). "Quantitative Muscle Ultrasonography Using 2D Textural Analysis: A Novel Approach to Assess Skeletal Muscle Structure and Quality in Chronic Kidney Disease." Ultrason Imaging **43**(3): 139-148.
Wilkinson, T. J., L. A. Baker, E. L. Watson, K. Nikopoulou, C. Karatzaferi, M. P. Graham-Brown, A. C. Smith and G. K. Sakkas (2024). "Skeletal Muscle Texture Assessment Using Ultrasonography: Comparison with Magnetic Resonance Imaging in Chronic Kidney Disease." Ultrason Imaging **46**(4-5): 263-268.
Zadeh, F. S., R. G. L. Koh, B. Dilek, K. Masani and D. Kumbhare (2023). "Identification of Myofascial Trigger Point Using the Combination of Texture Analysis in B-Mode Ultrasound with Machine Learning Classifiers." Sensors (Basel) **23**(24).
